# RDE & dynamometer analysis of light-duty vehicle emissions across altitudes, temperatures, and driving styles

**DOI:** 10.1371/journal.pone.0318298

**Published:** 2025-03-19

**Authors:** Jiguang Wang, Li Wang, Jiaqiang Li, Jianwei Li, Feng Xu, Fei Han, Jianliang He, Qiuling Chen, Xudong Chen

**Affiliations:** 1 CATARC Automotive Test Center(Kunming) Co., Ltd., Kunming, China; 2 China Automotive Technology and Research Center Co., LTD, Tianjin, China; 3 School of Mechanics and Transportation, Southwest Forestry University, Kunming, China; Vietnam Maritime University, VIET NAM

## Abstract

This study aimed to investigate the impact of altitude, cold start, ambient temperatures, and driving behaviors on the Real Driving Emissions (RDE) of China VI standard light-duty gasoline vehicles. Tests were conducted on actual roads and in vehicle emission environment simulation laboratories at altitudes of 700 m, 1,300 m, 1,900 m, and 2,400 m in Yunnan. The results showed that: (1) as altitude increased, the CO emission factor exhibited a positive correlation trend, peaking at 2400 m with a 2.56-fold increase compared to 700 m. The NO_X_ emission factor displayed an “N” distribution, with aggressive driving at 1900 m yielding 1.89 times higher emissions than normal driving and 3.02 times higher than low-temperature driving; (2) under low-temperature rotating wheel conditions, PN emission factors were 7.55 times higher than normal driving and 71.71 times higher than aggressive driving, indicating that driving behavior significantly influenced NO_X_ emissions, while low-temperature environments had a greater impact on PN emissions; (3) compared to non-cold-start conditions, the cold-start phase increased urban CO, NO_X_, and PN emission factors by 4.72% to 225.00%, 0.19% to 15.38%, and 6.45% to 430.36%, respectively, with total emission factors increasing by 0.07% to 0.55%, 0.00% to 6.00%, and 1.03% to 242.64%.

## Introduction

As the road vehicle with the largest production, sales, and ownership of motor vehicles in China, light-duty vehicles have the characteristics of large total amounts of pollutants and carbon emissions and wide operating areas. They have become the focus of China’s control in the fields of motor vehicle pollution reduction and carbon reduction. However, some studies have shown that laboratory test cycles cannot fully reflect the actual driving conditions [1–4], resulting in large differences between laboratory emission results and actual emissions [5–8].

In order to accurately test and evaluate the actual road pollutant emission characteristics of vehicles, the China Ⅵ emission standard for light-duty vehicles (GB18352.6-2016) includes RDE into the type approval content for the first time [9]. Many scholars use the Portable Emission Measurement System (PEMS) to conduct actual road emission tests on traditional light-duty gasoline and diesel vehicles and hybrid vehicles [10–16]. Different from the strict boundary condition control in laboratory test, the RDE test allows expansion in the ambient temperature range of −7 ~ 35 °C and the altitude range of 0 ~ 2400 m and conducts actual road emissions on actual roads composed of urban, suburban, and highway, and use the CO_2_ moving average window method (MAW) to calculate emissions [17]. With the implementation of China VI standards, a lot of scholars and institutions have focused on the boundary conditions that may affect RDE test results, including stroke dynamics parameters [18–20], altitude [21–23], cold start [24,25], data treatment methods [26–28] and road gradient conditions [29] have been studied. Related factors have an impact on the actual road emissions of light-duty vehicles. Roberto Aliandro Varella et al. [30,31] research team conducted extensive RDE tests on light-duty vehicles, employing MAW, PB, and VSP methodologies to process the data. They observed that while overall CO_2_ and NO_X_ emissions were comparable among the methods, notable differences emerged in NO_X_ emissions specifically during urban driving. The PB and VSP methods, due to their similar theoretical foundations, exhibited similar trends in CO_2_ and NO_X_ emissions, with a strong correlation. Furthermore, the study introduced the concept of emissions index to analyze pollutant emissions during cold starts, revealing that lower ambient temperatures led to increased NO_X_ emissions, with a more pronounced effect on gasoline-powered vehicles.

Separately, Kwon S et al. [32,33] and his team utilized PEMS equipment to test light-duty diesel vehicles under RDE conditions, uncovering that NO_X_ emission factors varied significantly (−4% to 60%) depending on the driving route. Notably, the PB method yielded lower NO_X_ emission factors compared to the MAW method. Jens Gallus et al. [34,35] compared PN emissions between laboratory bench tests and real-world driving conditions, finding a high degree of consistency but also significant variations attributed to changing driving conditions. Additionally, they observed a sharp increase in PN emissions during cold starts under low temperatures. Research into driving behavior indicated that aggressive driving significantly elevated CO_2_ and NO_X_ emissions, with CO_2_ increasing by 20–40% and NO_X_ by 50–255%, while CO and HC emissions showed lesser variation. The Polish BOSMAL Automotive R & D Institute, led by Piotr Bielaczyc et al. [36] investigated the emission characteristics of gasoline and diesel vehicles under different test cycles (NEDC, WLTC, FTP-75). They found that NO_X_, PM, and PN emissions were significantly higher under WLTC conditions. While NEDC and WLTC showed relatively similar emission compliance factors, NEDC and FTP-75 displayed greater discrepancies. Moreover, Piotr Bielaczyc’s et al. [37] research emphasized that RDE conditions offered a broader load distribution compared to NEDC. WLTP conditions produced CO_2_ emission factors that were on average 10% higher than NEDC but closer to RDE. However, WLTP’s NO_X_ emission factors were 2–5 times higher than NEDC, and RDE’s NO_X_ emissions were approximately three times higher than WLTP. CADC’s NO_X_ emission factors were similar to RDE, while DYN’s were 2–8 times higher. Collectively, these studies underscore the complexity and variability of vehicle emissions, emphasizing the crucial role of testing methodologies and driving conditions in accurate emission assessments.

However, due to the poor reproducibility of RDE testing on actual roads conditions, there are still considerable differences in conclusions. In addition, the China Ⅵ standard stipulates that the RDE test only records cold-start emission data, which needs to be excluded from the final urban and total emission calculations, resulting in the calculation results not fully reflecting the actual road emission situation.

As the major source of air pollutants. light-duty vehicles contribute more to the world’s air pollution than any other human activity, especially in high-traffic areas [38]. Light-duty gasoline vehicles are responsible for almost all of the carbon monoxide and lead in urban air, as well as most of the nitrogen oxides, volatile organic compounds (VOCs), particulates and toxic chemicals [39]. As the world’s leading consumers of oil, vehicles also emit large amounts of carbon dioxide and other gases that contribute to global warming. Vehicle also play a major role in stratospheric ozone depletion due to the widespread use of chlorofluorocarbons (CFCs) in automobile air conditioning. The damage caused by vehicle pollutants has become unavoidable. Although almost 50% of all new vehicles produced each year are equipped with emission control devices, the number of vehicles and the number of vehicle miles travelled are growing much faster than the emission reductions that have been achieved to date [40]. Research on light-duty vehicle emission characteristics is critical to saving energy and reducing carbon emissions.

Cold-start emissions account for a large proportion of urban trip emissions, and the effect of driving behavior on cold-start emissions is more significant and sensitive than the effect on non-cold-start emissions in RDE tests [41]. Based on RDE tests conducted at different ambient temperatures, it was also shown that the effect of ambient temperature on cold-start emissions is only a minor factor of driving behavior [42]. To address the effect of high altitude on vehicle emissions, the China VI emission standards introduced the RDE test program. The results showed that carbon monoxide emissions increased with altitude. PN and NO_X_ emissions also increased with altitude increasing, while NO_X_ emissions at 2990 m showed a decreasing trend. In general, the higher altitude, the lower the vehicle CO_2_ emissions. The CO_2_ emissions of the naturally-aspirated test vehicles correlate linearly with altitude: for every 1,000 m of altitude gain, the WLTC CO_2_ emissions decrease by 5.31% [43]. Not only does the intensity of the driving maneuver have an effect on the emission factors, but also the duration and frequency of individual operating states [44].

This article selects a light-duty gasoline vehicle that meets the China VI Standards Stage B as the research object and uses the PEMS to test actual road emissions at altitudes of 700 m, 1300 m, 1900 m, and 2400 m. At the same time, CATARC Automotive Test Center (Kunming) Co., Ltd. (referred to as Kunming Test Center) have built a light-duty vehicle emission environment simulation laboratory at the actual altitude of 1900 m in Kunming City to carry out Type I te0sts (exhaust pollution after cold start at normal temperature emissions test) and RDE tests based on vehicle dynamometers, study and analyze the influence of cold start, altitude, ambient temperature and driving behavior (dynamic parameters) on RDE emissions. The aim of this paper is to investigate the emission characteristics of China VI light-duty gasoline vehicles under road conditions at different altitudes in Yunnan Province. In this paper, we conducted RDE tests in four cities with different altitudes in Yunnan Province. We used the moving average window method to analyze the RDE test data to compare the effects of altitude, cold start, ambient temperature, and driving behavior on the RDE test. The conclusions of this paper can shed light on the emission characteristics of China VI light-duty gasoline vehicles under different conditions, provide useful information for policy formulation, and provide a reference for the improvement of RDE regulations.

This study conducted RDE tests on light-duty gasoline vehicles complying with China’s National VI standards in different altitude regions of Yunnan Province, China, at altitudes of 700 m, 1300 m, 1900 m, and 2400 m. The tests were carried out on actual roads and in vehicle emission simulation laboratories. This study reveals new insights into the emission characteristics of these vehicles under various environmental conditions, with its main novelty lying in the comprehensive investigation of the combined effects of altitude, cold start, ambient temperature, and driving behavior on vehicle emissions.

The main objectives of this study are as follows:

(1) To assess the specific impact of altitude changes on the emission factors of CO, NO_X_, and PN;
(2) To thoroughly analyze the impact of different driving behaviors (including aggressive driving, normal driving, and low-temperature driving conditions) on NO_X_ and PN emissions;
(3) To compare vehicle emissions during the cold start phase with those under urban driving conditions, in order to fully understand the combined effects of different driving phases and environmental conditions on vehicle emissions.

## Materials and methods

### Test vehicle

A light-duty gasoline truck, which meets the China VI Standard Stage B, was selected as the research object. It is equipped with a 1.5 L naturally aspirated engine, and the after-treatment system used a three-way catalytic converter. During the testing process, the test vehicle used the same batch of commercially available #92 gasoline that meets China VI standards, driven by a professional RDE driver and accompanied by a researcher who monitored the data in real-time. The main technical parameters of the test vehicle are shown in Table 1.

**Table 1:** Main technical parameters of the test vehicle.

Item	Parameters
**Air Intake method**	Inhale naturally
**Engine capacity/L**	1.48
**Curb weight/kg**	1620
**Gearbox type**	5MT
**Engine rated power(kW)/Rotating speed(r/min)**	80/6000
**Engine peak torque(N/m)/Rotating speed(r/min)**	150/4000
**Aftertreatment system**	TWC+TWC
**Mileage/km**	318
**Year of manufacturing**	2023
**Type of vehicle**	Light-duty truck
**Loading**	40%
**Lambda parameter**	14.7±1

**Fig 1:**
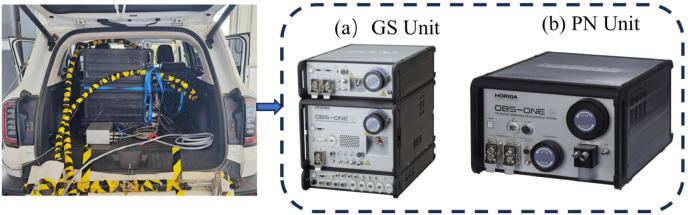
OBS-ONE portable emission measurement system.

Fig 1 shows the installation diagram of the vehicle emission testing equipment. The OBS-ONE [45] manufactured by HORIBA, Japan, was selected, which consists of two main modules, i.e., the gas module, the particulate module. The measurement parameters of the main pollutants in OBS-ONE are shown in Table 2. The gas module is used to test the concentration of gaseous pollutants in the vehicle exhaust and consists of a gaseous pollutant analyzer (OBS-ONE GS), which uses non-dispersive infrared (NDIR) to measure CO and CO_2_, and chemiluminescent detector (CLD) to measure NO and NO_2_. In the particulate module, the on-board measurement system (OBD) is used to analyze the gaseous pollutant concentration in the vehicle exhaust by condensation particle CPC (Condensation Particle Counter) method to analyze the number concentration of solid particles in a specified particle size range. The test system is also equipped with a Global Positioning System (GPS) receiver that calculates vehicle speed based on the vehicle’s longitude, latitude and altitude. In addition, PEMS is equipped with an atmospheric pressure sensor (measuring range 0 to 115 kPa with an accuracy of ± 2% of full scale), an ambient temperature sensor (measuring range −40 °C to 60 °C with an accuracy of ± 0.5 °C) and a humidity sensor (measuring range 0 to 100 RH with an accuracy of ± 1.5% of full scale). Information about the environmental conditions during the test is obtained from these sensors. The test system also collects engine operating data through OBD, including engine speed, engine coolant temperature, throttle position, fuel consumption rate, TWC converter temperature, intake mass flow, air pressure and other data, and exhaust mass flow. The test system is powered by an external power supply at 24 V. Before use, the PEMS unit is warmed up and calibrated according to RDE test regulations.

**Table 2:** Measurement parameters of the main contaminants of OBS-ONE.

Pollutants	Analysis module	Measuring range	Measurement accuracy
**CO**	NDIR	0~10%vol	±0.3%
**CO**_**2**_	NDIR	0~20%vol	±0.3%
**NO,NO**_**2**_ **and NO**_**X**_	CLD	0~3000ppm	±0.3%
**PN**	CPC	0~5×107#/cm3	±10%

### Experiment with test boundary conditions

Based on the Yunnan Plateau mountainous three-dimensional road, an actual road condition emission test was carried out. During the test, the vehicle air conditioner was turned on, and the interior temperature was maintained within the range of 23 ± 2 °C. The influence of altitude, ambient temperature, and driving behavior on RDE emission results and control the errors of variables such as ambient temperature and road conditions during the test were studied. Ambient temperature can affect the performance of a vehicle’s engine [46]. Low temperatures can lead to decreased engine efficiency due to thicker oil and slower chemical reactions, while high temperatures may cause the engine to overheat, increasing wear on engine components [47]. These factors, in turn, can impact the vehicle’s ability to maintain a certain speed or achieve optimal fuel efficiency. Secondly, ambient temperature also affects the interior comfort of the vehicle. Extreme temperatures, both hot and cold, can make it difficult for passengers to maintain a comfortable body temperature. This can affect their comfort level and overall satisfaction with the vehicle’s performance [48]. In our experiments, we recognize that ambient temperature was not directly controlled or recorded. However, we took measures to ensure that the experiments were conducted under similar conditions, such as scheduling the tests during similar times of day or using temperature-controlled environments. While this may not fully eliminate the effects of ambient temperature, it helps minimize potential variations and ensures the consistency of our results. In future work, we plan to consider ambient temperature as a variable in our experiments. This will allow us to more thoroughly evaluate its effects on vehicle performance and interior comfort, and provide more comprehensive recommendations and insights.

The influence of humidity on engine characteristics is a complex and multifaceted phenomenon, particularly during road testing, where the effects become more pronounced due to varying weather conditions such as rain, snow, or fog. As humidity increases, the water vapor content in the air also rises, leading to a decrease in air density [49]. For engines, this reduced air density means a reduced volume of air entering the cylinders per unit time, affecting the fuel-to-air mixture ratio and subsequent combustion process. Specifically, increased humidity may result in condensation of water within the engine’s intake system, forming droplets or mist [50]. This can obstruct the intake passages, reduce airflow, and potentially cause corrosion in the combustion chamber. Additionally, the water content in the mixture absorbs heat, lowering the combustion temperature and reducing the combustion speed, which affects the engine’s power output and fuel economy [51]. During rainy, snowy, or foggy weather conditions, engines face even greater challenges during road testing. Rainwater not only increases humidity but can also splash directly into the engine’s intake or exhaust passages, causing malfunctions or damage. Snowy and foggy days may also impact the engine’s heat dissipation performance, leading to overheating or performance degradation [52].

**Fig 2:**
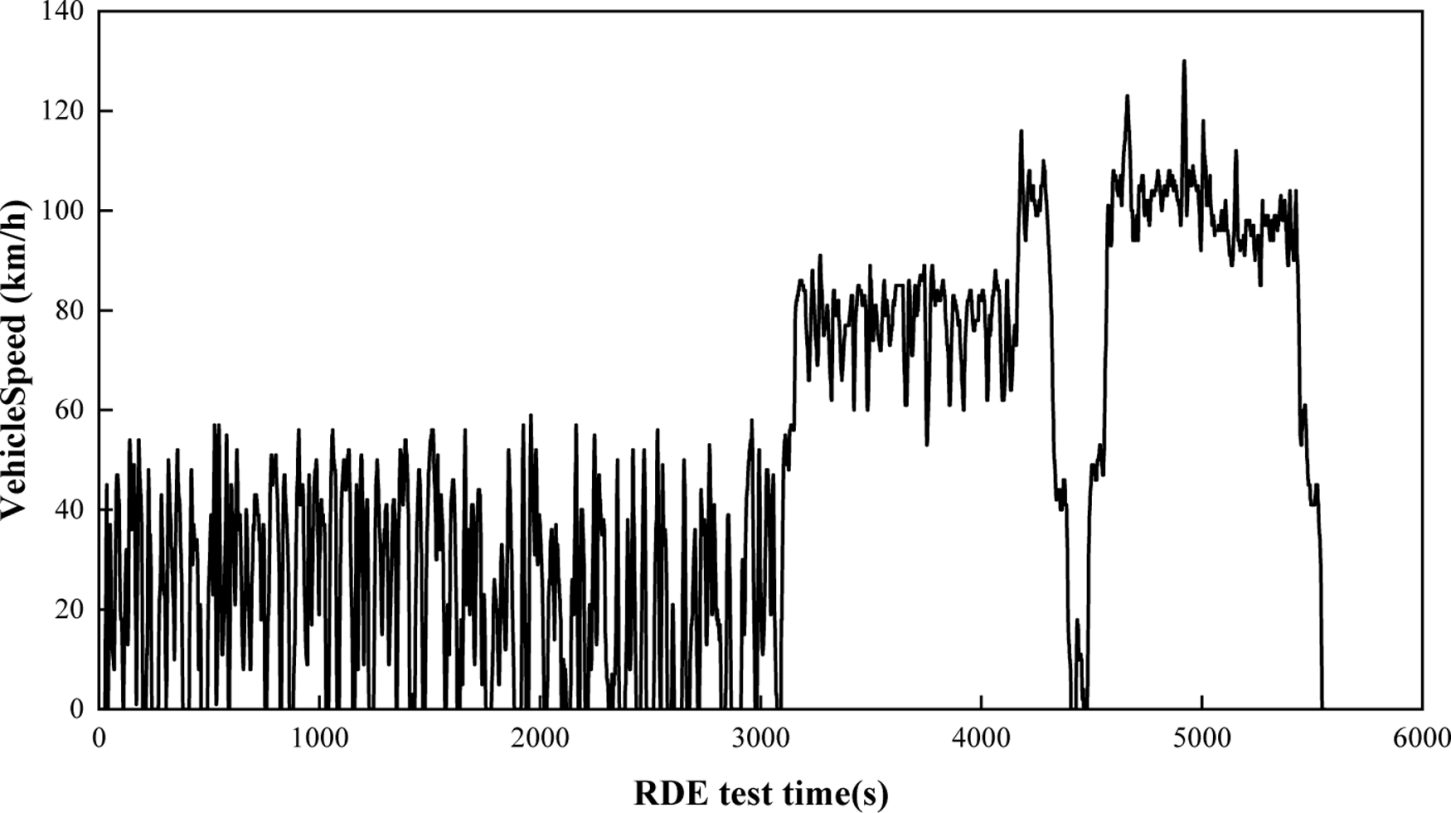
Vehicle speed cycle of RDE.

The test is conducted in strict accordance with the requirements of the RDE regulations, the duration of the RDE test is between 90 min–120 min, the driving route includes 34% of the urban road, 33% of the suburban road, 33% of the highway road, and the each road section proportion of the driving error is controlled within 10%, the minimum distance of each section of the driving is greater than 16 km, and the difference in altitude between the test start point and the end point is less than 100 m. Fig 2 shows the vehicle speed cycle of RDE, which reflects the characteristics of actual road conditions in Yunnan province and meets the GB18352.6-2016 standard requirement. In order to reduce the error of test results, two tests were conducted under each altitude condition, and the moving average window method was used for calculation. After the pollutant deviation of the two tests was ≤10%, the arithmetic mean of the two test results was taken as the final test. The results are analyzed; otherwise, additional tests are required for testing. Table 3 shows the RDE test location and altitude. The altitude covers ordinary altitude conditions, extended altitude conditions and further extended altitude conditions.

**Table 3:** Experimental test schemes.

No.	Test locations	Altitude (m)	Driving behavior	Test site	Ambient temperature(°C)	Atmospheric pressure (kPa)	Test mileage (km)
A	Xishuangbanna	660~735	Normal driving	Road testing	20±2	93.9	74.45
B	Jianshui	1300±50	Normal driving	Road testing	27±3	86.8	65.84
C	Kunming	1900±50	Normal driving	Road testing	23±3	80.4	80.34
D			Aggressive driving	Road testing	22±3	80.5	76.76
E			Normal driving*	Dynamometer testing	-5±-2	81.8	78.72
F	Lijiang	2400±50	Normal driving	Road testing	9±2	77.2	66.27

*:The same speed and slope coupling condition curve as C is adopted.

## Analysis of test results

### RDE test condition judgment and verification

#### Judgment of window integrity and normality.

The RDE test standard stipulates that the normality and integrity of the window should be verified when calculating using the moving average window method. The integrity verification requires that the number of windows in urban, suburban, and highway sections be greater than 15% of the total number of windows, and the integrity of the six RDE experiments all passed the requirements [53].

**Fig 3:**
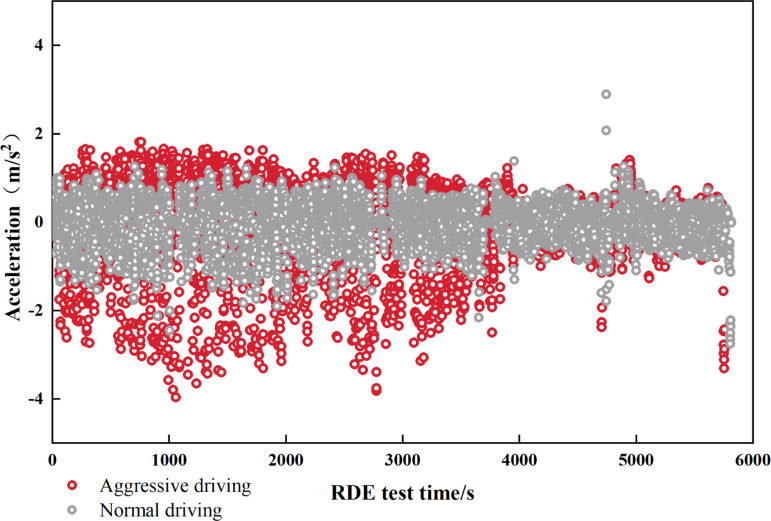
Distribution of acceleration conditions under aggressive driving and normal driving at an altitude of 1900 m.

In the calculation of the CO_2_ MAW method, the “vehicle - CO_2_ characteristic curve” needs be used to evaluate the normality of the CO_2_ window. The vehicle CO_2_ characteristic curve is shown in Fig 3. The irregular curves in the figure are obtained from the RDE test and correspond to the CO_2_ window on the axes. The parameters at points P1, P2, and P3 consist of dots, which are determined by the average speeds and CO_2_ emission factors in the low-speed, high-speed, and ultra-high-speed segments of the vehicle’s WLTC cycle. The three points are connected to form the CO_2_ characteristic curve of the vehicle. The window average speed is bound by 45 and 80 km/h and is divided into urban, suburban, and highway. The basic tolerance and extended tolerance of the vehicle CO_2_ characteristic curve are defined as tol1 = 25% and tol2 = 50%, respectively.

The RDE test procedures require that when more than 50% of urban, suburban, and highway windows fall within the basic tolerance range defined by the characteristic curve, the results can be judged to be normal. If the above minimum requirement of 50% is not met, the range of the upper limit tol1 can be increased in steps of 1% until the 50% window requirement is met [36]. Table 4 shows the normality and completeness of the CO_2_ window of the test prototype, both of which meet the requirements of the GB18352.6-2016.

**Table 4:** Normality and integrity of CO_2_ window.

No.	Normality of CO_2_ window			Integrity of the CO_2_ window			Expanding tolerances tol_1_/%
	Urban/%	Suburban/%	Highway /%	Urban/%	Suburban/%	Highway /%	
**A**	100.0	92.6	100	46.1	36.9	16.9	25
**B**	100.0	99.5	98.9	51.5	25.3	23.2	25
**C**	100.0	83.0	74.4	40.5	39.3	20.2	25
**D**	50.5	100.0	100.0	49.3	29.2	21.5	32
**E**	94.7	100.0	100.0	41.9	37.9	20.2	25
**F**	100.0	100.0	100.0	53.3	27.0	19.7	25

#### Verification of stroke dynamics parameters.

The GB18352.6-2016 standard stipulates that RDE testing requires verification of stroke dynamic characteristics to determine whether all dynamic characteristics are excessive or insufficient while driving in urban, suburban, and highway sections [54]. The core of stroke dynamics verification lies in two parameters: RPA (relative positive acceleration) and v.a_pos-_[95] (95th percentile of the product of vehicle speed and positive acceleration greater than 0.1 m/s^2^), divided according to instantaneous speed per second. The three-speed sets (urban, suburban and highway) all need to meet the verification of two parameters, respectively, and the RDE test trip is considered valid. RPA is used to characterize the actual road driving acceleration and intensity of vehicle [55]. The RDE test requires verification of the RPA of each speed group to deter-mine whether the driving stroke is too gentle. The purpose of verifying the RPA is to ensure that the driving is not too gentle.


$$\text{RPA}=\frac{\int_{0}^{\text{T}}�V_{i}\times a_{i}^{+}�\cdot dt}{x}$$ (1)

In the formula, a_i_^ +^ is the positive acceleration of the test vehicle in i second, m/s^2^;V_i_ is the speed of the test vehicle in i second, m/s; X is the displacement of the test vehicle within the time interval T.


$${\text{a}}_{i}=\frac{V_{i+}{}_{\Delta t}-V_{i-}{}_{\Delta t}}{2\Delta t}$$ (2)

In the formula: a_i_ is the acceleration of the test vehicle in i second, m/s^2^; Δt is the time interval between *i* second and Δ*i* + t, where Δt = 1, s.

v·a_pos-_[95] represents the 95th percentile value of vehicle acceleration within a specific time period or on a particular road section. It signifies that 95% of all the collected acceleration data are less than or equal to this specific value. This parameter can intuitively reflect the intensity of acceleration behavior during the vehicle’s actual driving process [54]. Specifically, a higher v·a_pos-_[95] value usually indicates more aggressive driving behavior, such as frequent acceleration or hard acceleration. The purpose of verifying v.a_pos-_[95] is to limit driving from being too aggressive. v.a_pos-_[95] in each speed group should be verified during RDE testing.

The RDE standard stipulates that the stroke dynamic parameters such as acceleration, a_pos_ (positive acceleration greater than 0.1 m/s^2^), and RPA are calculated based on the speed signal with a vehicle speed greater than 3 km/h, an accuracy of 0.1%, and a sampling frequency of more than 1 Hz. The v.a_pos-_[95] and RPA verification results of the test vehicle under different altitude conditions are shown in Table 5.

**Table 5:** v.a_pos-_[95] and RPA verification for each RDE segment.

No.	v.a_pos-_[95](m^2^/s^3^)			RPA(m/s^2^)			Verified results
	Urban	Suburban	Highway	Urban	Suburban	Highway	
**A**	7.6 (17.561)	14.0 (24.388)	11.0 (26.444)	0.2036 (0.139)	0.1652 (0.059)	0.0664 (0.025)	Passed
**B**	8.6 (17.383)	14.9 (24.327)	16.7 (26.483)	0.1999 (0.141)	0.1824 (0.059)	0.1354 (0.025)	Passed
**C**	8.0 (17.953)	12.3 (24.647)	13.6 (26.425)	0.2247 (0.134)	0.1585 (0.035)	0.1160 (0.025)	Passed
**D**	12.4 (17.695)	15.6 (24.752)	13.6 (26.273)	0.4337 (0.137)	0.2278 (0.051)	0.1167 (0.025)	Passed
**E**	6.9 (17.834)	11.0 (24.563)	10.3 (26.336)	0.1758 (0.136)	0.1112 (0.055)	0.0677 (0.025)	Passed
**F**	7.5 (17.322)	16.9 (24.069)	21.2 (26.065)	0.2600 (0.142)	0.1900 (0.062)	0.1070 (0.025)	Passed

It can be seen from Table 5 that the actual values of v.a_pos-_[95] of each road section of the test sample under different altitude conditions are less than the reference values in the brackets, and the actual RPA values of each road section are greater than the reference values in the brackets. RDE test both v.a_pos-_[95], and RPA have passed the verification. In the stages of urban, suburban, and highway, the v.a_pos-_[95] and RPA of test condition D are significantly higher than other normal driving test conditions. From this, it can be seen that the intensity of aggressive driving in test condition D is more obvious. In addition, under test conditions A to F, RPA gradually decreases on urban, suburban, and highway roads, which shows that the actual intensity of vehicle driving on roads gradually decreases and is also consistent with the vehicle driving speed characteristics of different roads.

Fig 3 shows the acceleration characteristic distribution during aggressive driving and normal driving during the RDE test at an altitude of 1,900 m. It can be seen from Fig 2 that the acceleration distribution of normal driving is mainly concentrated in the range of −1.5~1 m/s^2^, while the acceleration of aggressive driving is mainly concentrated in the range of −3~2 m/s^2^, which is significantly higher than normal driving. At the same time, aggressive driving conditions are mainly reflected in urban and suburban road sections. The acceleration distribution range is large, especially since the deceleration process is more aggressive. The deceleration under extreme driving conditions reaches −4 m/s^2^, while in high-speed sections, it is controlled by the speed range of the highway. At limits such as 80~120 km/h, the difference in acceleration distribution characteristics between normal driving and aggressive driving is very small. From this, it can be inferred that aggressive driving conditions mainly have a greater impact on emissions in urban and suburban stages [56].

The statistical analysis of RDE tests and bench tests.

In order to further analyze the difference between the emission results of the dynamometer and actual road conditions, the test vehicle in the study was carried out the WLTC emission test by dynamometer under the 23 °C normal temperature and −7 °C low temperature environment and the altitude of 1914 meters, respectively. The results were compared with the test results of No. C condition and No. E condition respectively, as shown in Table 6.

**Table 6:** The statistical analysis of RDE tests and bench tests.

Test condition		CO(mg/km)	NO_X_(mg/km)	PN(#/km)
**23°C** **Normal temperature condition**	**Dynamometer test** **Altitude 1914m,WLTC**	131.20	5.93	2.13×10^10^
	**No. C condition**	164.00	8.00	2.45×10^10^
**-7°C** **Low temperature condition**	**Dynamometer test** **Altitude 1914m,WLTC**	199.47	11.46	3.14×10^10^
	**No. E condition**	269.28	15.13	3.92×10^10^

The results show that the pollutant emission results of CO, NO_X_ and PN in No. C condition are about 1.25 times, 1.35 times and 1.15 times of that in WLTC condition at 23 °C normal temperature, respectively. The same pollutant emissions in No. E condition are about 1.35 times, 1.32 times and 1.25 times of that in WLTC condition, respectively. There are two main reasons, First, the large difference between the WLTC condition and the actual road condition, especially the WLTC condition is only a “Speed-Time” relationship curve, while the actual road slope and other road factors have a significant impact on emissions. Second, the actual road test results are affected by driving behavior, environmental temperature and humidity [46], and the test conditions are more complex, resulting in the actual road emissions are significantly higher than the dynamometer test results [48].

### Analysis of emission factors of test vehicles at different altitudes and under road conditions

**Fig 4:**
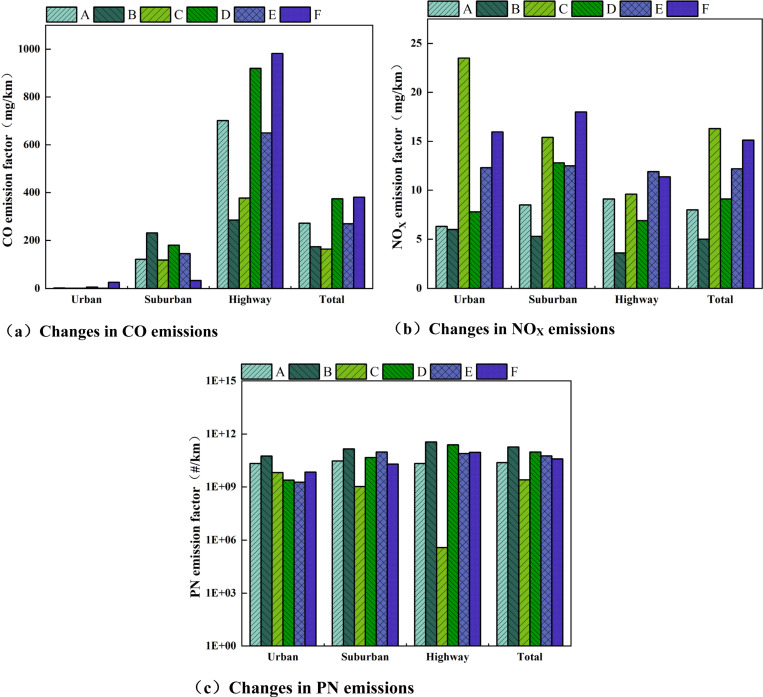
Changes in conventional pollutant emissions at different altitudes at different stages.

In order to study the pollutant emission characteristics of different test roads in the RDE test, the pollutant emission analysis is divided into four stages: urban, suburban, high-speed, and total. Under comprehensive test conditions such as different altitudes, temperatures, and driving behaviors, the emission factors of three pollutants, CO, NO_X_, and PN, of the test vehicle in urban areas, suburbs, highways, and the entire actual road are shown in Fig 4, and Table 7 shows the explanation of A-F. Overall, as the altitude increases, there are certain differences in the emission trends of CO, NO_X_, and PN pollutants from test vehicles in various road sections [57].

**Table 7:** The meaning of A-F.

**A**	700m
**B**	1300m
**C**	1900m (Normal driving)
**D**	1900m (Aggressive driving)
**E**	1900m (-7°C)
**F**	2400m

Fig 4(a) shows that the CO emission factor is positively correlated with the in-crease in altitude in urban and suburban areas and total travel stages. The CO emission factor is the largest when the altitude reaches 2400 m. Compared with the altitude of 700 m, the CO emission has increased about 2.56 times. Analyzing the main reasons, this paper studies the test vehicle equipped with a 1.5 L naturally aspirated engine. Com-pared with the plain environment, light-duty vehicle engines with naturally aspirated technology have reduced intake pressure, airflow, and insufficient engine power in the plateau region, resulting in increased injection penetration distance and weakened engine combustion performance, especially in urban areas under low-load conditions [58]. Therefore, enrichment will occur under medium and large loads in suburban areas and highways. At this time, the altitude factor has weakened; the CO emissions at an altitude of 1900 m are only about two times higher than those at an altitude of 100 m. During the urban start-up stage, in order to ensure the normal start of the vehicle, the fuel injection volume is relatively large, but the reduction in air intake volume causes the mixture to be (locally) too rich, exacerbating CO emissions. CO emissions in the high-speed stage first decrease and then increase with increasing altitude. Especially under aggressive driving behavior, the CO emissions in the high-speed stage rise instantly. At this time, the engine load is large, the cylinder fuel injection volume in-creases, and the probability of insufficient fuel combustion increases, making it difficult for the fuel in the cylinder to reach the optimal air-fuel ratio in a short period of time, resulting in insufficient combustion of the mixture and causing an increase in CO emissions from vehicles in the high-speed segment at high engine speeds and high loads.

NO_X_ emissions mainly come from transportation (especially road transportation and shipping), oil and gas activities, and land-based industries. NO_X_ refers to com-pounds composed of only two elements, nitrogen and oxygen [59]. Fig 4(b) shows that the full NO_X_ emission factor shows an “N” type change trend as the altitude increases from 700 m to 2400 m. At an altitude of 2400 m, the NO_X_ emission factor is the highest, reaching 16.30 mg/km. However, it is worth noting that at an altitude of 1900 m, com-pared with normal driving (C) and low-temperature hub (E) conditions, the NO_X_ emission factor under aggressive driving (D) conditions is significantly higher, which is approximately 1.89 times of the C driving conditions, and 3.02 times that of driving conditions E. It can be seen that the intensity of driving behavior has a greater impact on NO_X_ emissions. Analyze the reasons: NO_X_ generation conditions are high-temperature and oxygen enrichment, drastic load changes during aggressive driving behavior, a continuous high-temperature environment, and obvious increase in transition conditions, and other factors that lead to an increase in engine fuel injection and a significant increase in NO_X_. Under various altitude conditions, the NO_X_ emission factor in the urban stage is higher than that in the suburban and highway stages, mainly because NO_X_ is generated under high temperatures and oxygen-rich conditions. In the urban stage, fuel injection is enriched to ensure vehicle power under medium and high loads, making it difficult to generate NO_X_; fuel injection and enrichment are not performed under low-load conditions in urban areas, so NO_X_ emissions are relatively small. The overall change in NO_X_ increases with the increase in altitude, and the NO_X_ emission shows a trend of first decreasing and then increasing.

Fig 4(c) shows that the PN emission factor shows a “V” shaped trend with the increase in altitude from 700 m to 2400 m. However, compared to the normal driving (C) and aggressive driving (D) conditions at an altitude of 1900 m, the PN emission factor was the highest in the low-temperature rotating hub (E) condition at 1.85 × 10 + 11#/km, which is about 7.55 times of the driving condition C, and 71.71 times of driving conditions D. It can be seen that the low-temperature environment has a greater impact on PN emissions. Analyze the reasons: First, the engine cylinder wall temperature is low in low-temperature environments, and the mixture is not fully burned, which leads to an increase in soot, soluble organic matter, and other particles under low load. At this time, the TWC has not reached its ignition temperature, and the soluble organic matter has not been released in time. Oxidation leads to an increase in particulate matter emissions; second, PN emissions are related to the fuel oil film participating in combustion in the engine cylinder. The greater the quality of the oil film, the greater the PN emissions. The main reasons for the increase in oil film quality are: the higher the altitude, the lower the air density, the atmospheric pressure increases, the fuel spray penetration distance in-creases, and the oil film will be generated on the combustion chamber wall due to the spray hitting the wall. The higher the altitude, the greater the fuel penetration distance, the greater the quality of the coanda oil film, and the greater the impact on PN emissions. In addition, under different altitude conditions, PN emissions are mainly in the high-speed stage. The main reason for this is that the increase in fuel injection following the increased power output demand of the engine in the high-speed phase even causes the emission control strategy to open the cycle, but the oxygen content in the air decreases, leading to a sharp increase in particulate matter emissions.

### Analysis of the impact of cold start on pollutant emissions of light-duty vehicles

#### Effect of the cold start phase on pollutant emissions.

The GB18352.6-2016 standard stipulates that two conditions, time and engine coolant temperature, are used to determine whether the vehicle is in the cold start stage, that is, within 300 seconds after the vehicle starts the engine for the first time or the coolant temperature reaches 70 °C as a sign of the end of the cold start, but the premise is that the time for the coolant temperature to reach 70 °C does not exceed 300 seconds after the engine is firstly started [60].

The RDE emission test results are mainly calculated by multiplying the pollutant emission factors in three stages: urban, suburban, and highway, respectively, by weighting factors of 0.34, 0.33, and 0.33. The average coolant temperature during the cold start phase of the vehicle is mainly related to the initial coolant temperature (usually consistent with the ambient temperature) and the operating conditions of the engine. The low-temperature environment will affect the average coolant temperature and the ignition time of the exhaust after-treatment system and prolong the cold start [61]. Du-ration and thus have an impact on cold start emissions. However, cold start pollutant emissions are only generated during the starting process of the test vehicle, which only affects pollutant emissions in the urban phase and has no impact on the suburban and high-speed phases. Therefore, the study mainly analyzes the impact of a cold start on the urban phase and highway phase of the RDE test. The impact of pollutant emissions throughout the process [62].

**Fig 5:**
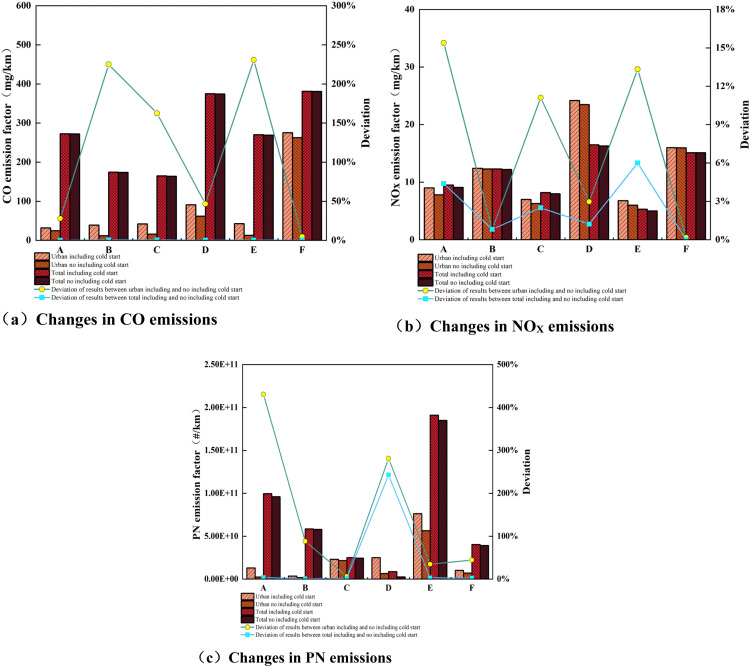
Comparison of pollutants in the urban phase and the whole process with and without cold start (a) CO emissions (b) NO_X_ emissions (c) PN emissions. “Deviation” refers to the difference in experimental results between the conditions where cold start is considered and where it is not.

Fig 5 shows a comparison of pollutant emission factors with and without the cold start phase for the city and full-scale tests, assessing and analyzing the impact of the cold start phase data on city and total pollutant emissions. It can be seen from Fig 4 that under various altitude test conditions, the pollutant emission factors in the urban and total stages with cold start data are higher than the results without cold start data, and the pollutant emission factors in the urban stage are higher than those without cold start data. The main reason is that the ignition time of the TWC of the test vehicle is about 100 seconds. The TWC of the test vehicle did not ignite after a cold start, and the catalytic conversion efficiency was low [63]. It was unable to efficiently reduce the emissions of various pollutants, resulting in an increase in pollutant emissions. Compared with the data without the cold start stage, the urban CO, NO_X_, and PN emission factors with the cold start stage increased by 4.72% ~ 225.00%, 0.19% ~ 15.38%, 0.19%~15.38% and 6.45%~430.36% respectively. The whole-process emission factors increased by 0.07% ~ 0.55%, 0.00% ~ 6.00%, and 1.03% ~ 242.64%, respectively. It can be seen from this that the cold start mainly has a greater impact on CO and PN emissions in the urban stage and a smaller impact on NO_X_ emissions, but has a greater impact on PN emissions in the entire stage.

#### Study on the transient emission characteristics of cold-start pollutants.

**Fig 6:**
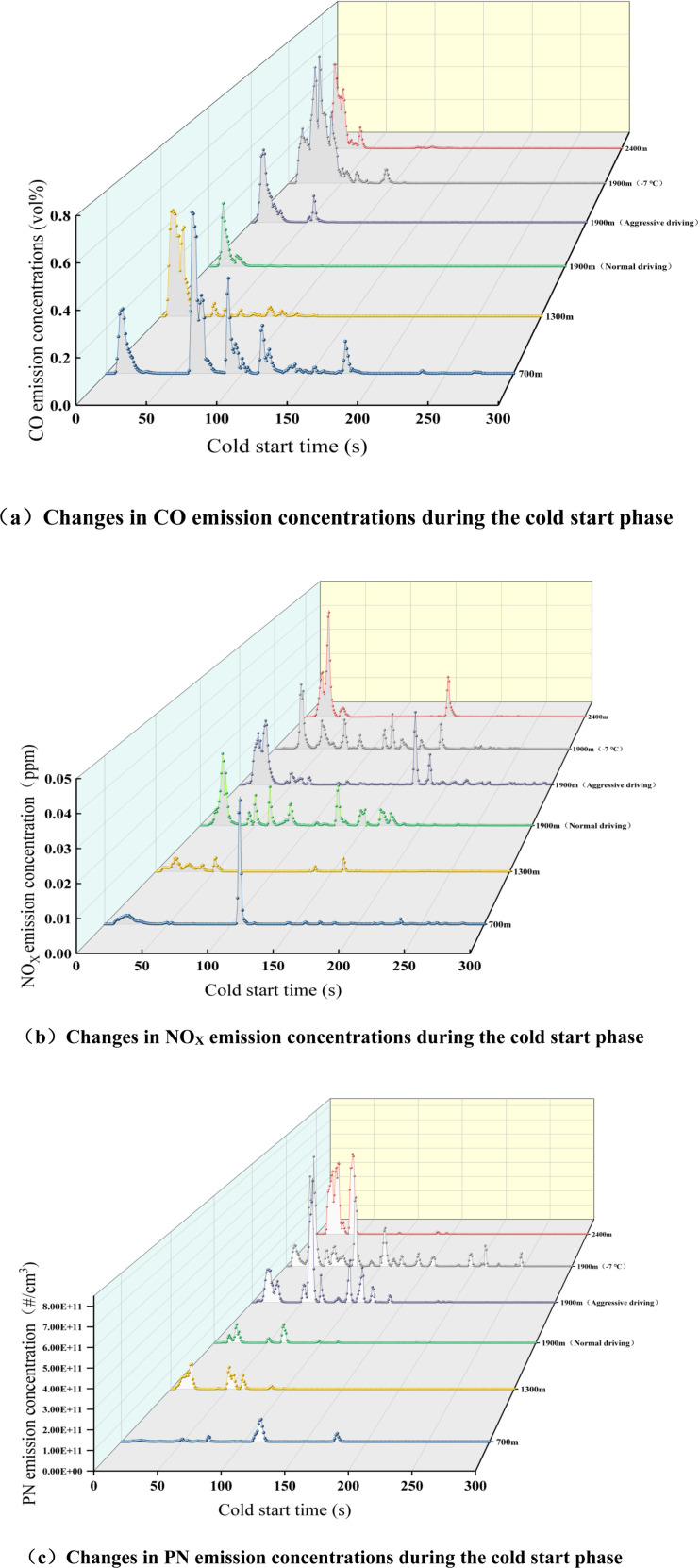
Conventional pollutant emissions during the cold start stage of automobiles at different altitudes (a) CO emissions (b) NO_X_ emissions (c) PN emissions.

The instantaneous emission data of CO, NO_X_ and PN within 300 seconds of engine cold start under different altitude conditions from A to F were selected to study and analyze the pollutant emission characteristics and change patterns of the test vehicle during the cold start stage [64]. As shown in Fig 6, there are certain differences between the instantaneous emissions of CO, NO_X_, and PN during the cold start stage of the test vehicle and factors such as altitude, driving behavior, and ambient temperature.

It can be seen from Fig 6(a) that as the altitude increases, the transient concentration of CO emissions shows an upward trend. Especially at the altitude of 2400 m, CO emissions increase sharply, and the peak emission concentration reaches 9%. The main reason is that as the altitude increasing, the atmospheric pressure, air density, and ambient temperature all decrease to varying degrees [65]. In order to start and run the engine smoothly, the ECU control system adopts an injection strategy of increasing the fuel injection volume, but the air intake volume of the low oxygen content leads to insufficient combustion in the engine cylinder and a significant increase in CO emissions [66].

It can be seen from Fig 6(b) that within 200 seconds after the engine is started, the transient concentration of NO_X_ emissions increases with the increase in altitude, especially when the peak value reaches 0.04 ppm at an altitude of 700 m. Since the air-fuel ratio in low-altitude areas is higher than that in high-altitude areas, the oxygen content in the mixture is sufficient, making it easier to generate NO_X_. In high altitude areas, the engine must operate under a larger load to obtain normal power output, which in turn leads to an increase in cylinder temperature and NO_X_ emissions. For aggressive driving condition D, the transient concentration of NO_X_ emissions suddenly increased sharply at 180 ~ 200 s during a cold start. The main reason was that the test vehicle accelerated sharply at this moment after the cold start, and the engine load increased significantly, resulting in a rapid increase in NO_X_ emissions. However, The TWC has not yet reached its ignition temperature and cannot effectively reduce NO_X_, resulting in an instant in-crease in the NO_X_ emission concentration in the exhaust gas [67].

It can be seen from Fig 6(c) that during the cold start phase, PN emissions are mainly generated in the first 100 s. The reason is that during the cold start phase of the test vehicle, the engine cylinder wall temperature is low, and the mixture is not fully burned. This leads to an increase in particles such as soot and soluble organic matter [68]. At this time, the TWC has not reached its ignition temperature, and the soluble organic matter is not oxidized in time, resulting in an increase in particulate matter emissions. In particular, the PN concentration was more obvious under the two test conditions of the aggressive driving test (D) at an altitude of 1,900 m and the low-temperature rotating hub test (E), with the peak concentration reaching 7 × 10 + 11#/cm3. The longer warm-up time of the engine under high temperature causes an increase in particulate matter emissions, while under aggressive driving conditions, the increase in the fuel injection volume of the test vehicle and insufficient in-cylinder fuel mixing and combustion leads to an increase in particulate matter emissions [69].

### Analysis of the impact of driving behavior on RDE emissions

**Fig 7:**
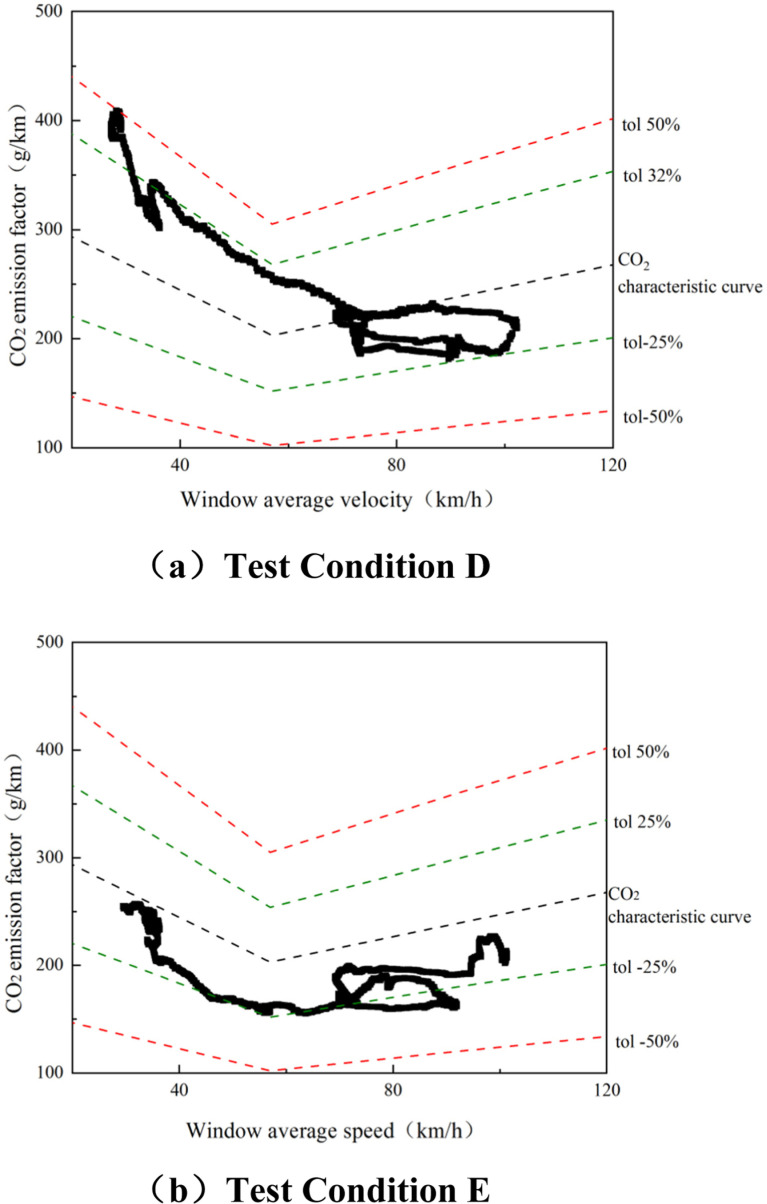
CO_2_ emission curves of normal and aggressive driving at an altitude of 1900 m.

In the study, two dynamic parameters, RPA and v.a_pos-_[95], were used to describe the driving behavior characteristics and the correlation between the dynamic parameters RPA and v.a_pos-_[95], and various pollutant emissions under different driving behaviors was analyzed. Fig 7 shows the comparison of CO_2_ emission characteristic curves under normal driving and aggressive driving conditions at an altitude of 1900 m. The thick black line represents the CO_2_ characteristic curve, which is part of the RDE window method verification.

It can be seen from Fig 6 that under normal driving conditions, the window CO_2_ emission factor is almost within the basic tolerance line (±25%) and is closer to the CO_2_ characteristic curve; under aggressive driving conditions, the window CO_2_ emission factor is Within the range of 25% ~ +50%, especially in the low and medium speed range where the vehicle speed is ≤ 80 km/h, the CO_2_ emission factor is within the range of the CO_2_ emission characteristic curve and the + 50% range. Comparing Fig 7a and Fig 7b, it can be seen that the change in CO_2_ emission factor is most obvious in the high-speed section (80 km/h < V < 120 km/h) window. Therefore, it can be inferred that urban and suburban roads have frequent acceleration and deceleration due to frequent vehicle acceleration and deceleration. The working conditions are more aggressive, and the aggressive driving operating point has a greater impact on the change range of the window CO_2_ emission factor.

### Analysis of the relationship between v.a _pos-_ [95] and pollutant emission factors

**Fig 8:**
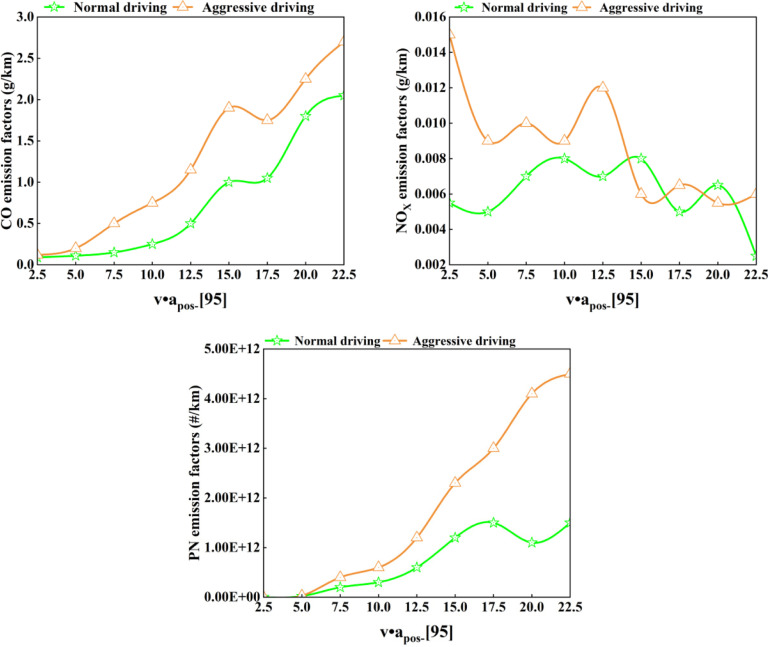
1900 m altitude at the relationship between normal driving and aggressive driving v.a_pos-_[95] and pollutant emission factors.

Fig 8 shows the relationship between v.a_pos-_[95] and pollutant emission factors under normal driving and aggressive driving conditions at an altitude of 1900 m.

It can be seen from Fig 7 that the CO and PN emission factors have a strong positive correlation with the intensity of actual road driving. Under the same altitude conditions, compared with normal driving, aggressive driving behavior causes CO emissions to increase by 33% to 235% and PN emissions to increase by 35% to 273%. The main reason is that drivers frequently drive violently during aggressive driving. When the accelerator pedal is stepped on, and the throttle is suddenly opened wide, the mixture may be momentarily lean due to the inertia of the mixture flow, the combustion temperature may be too low, and the mixture may be incompletely burned, etc., which may lead to increased emissions of CO and PN [70].

However, NO_X_ emissions do not show a good correlation with driving intensity, and there is no obvious pattern. When v.a_pos-_[95] is small, the more aggressive the driving, the higher the NO_X_ emission factor. The main reason is that during the vehicle’s starting stage, the internal temperature is low, and the TWC converter has not reached its light-off temperature, resulting in poor NO_X_ emissions. As v.a_pos-_[95] increases, the NO_X_ emission factors under normal driving and aggressive driving conditions gradually approach the same level. The reason for the above phenomenon is that when v.a_pos-_[95] has a large value, the vehicle is in a high-speed and high-load state. At this time, the gasoline engine valve opening is higher than about 85%. Due to the power mixture requirements, the engine needs to emit maximum power to overcome the larger External resistance, which requires the supply of a richer mixed gas. The excess air coefficient is generally 0.85 ~ 0.9. The mixed gas is incompletely burned, NO_X_ is easily generated under high temperature and oxygen-rich conditions, and its stoichiometric ratio deviates from the optimal range of TWC.

### Analysis of the relationship between RPA and pollutant emission factors

Table 8 shows the relationship between RPA and pollutant emission factors under normal driving and aggressive driving conditions at an altitude of 1900 m.

**Table 8:** RPA and pollutant emission factors under normal and aggressive driving conditions.

Area type	Aggressive driving				Normal driving			
	RPA (m/s_2_)	CO (g/km)	NO_X_ (g/km)	PN (#/km)	RPA (m/s_2_)	CO (g/km)	NO_X_ (g/km)	PN (#/km)
Urban	0.4337	1.6	23.5	6.6E+09	0.2247	6.2	7.8	2.5E+09
Suburban	0.2278	118.2	15.4	1.1E+09	0.1585	180	12.8	4.6E+10
Highway	0.1167	377.3	9.6	3.8E+07	0.116	920	6.9	2.4E+11

It can be seen from Table 6 that RPA has no obvious correlation with CO, NO_X_, and PN emissions. Under aggressive driving conditions, NO_X_ emissions have a strong positive correlation with RPA. As RPA increases, NO_X_ and PN emission factors show a significant increasing trend, reaching 23.5 g/km and 6.6 E + 09#/km, respectively, in urban areas, but the CO emission factor shows a downward trend with the increase of RPA. Under normal driving conditions, the CO emission factor also shows a downward trend with the increase of RPA, but the NO_X_ and PN emissions change irregularly with the increase of RPA. This also shows that compared with the dynamic parameters RPA, the correlation between v.a_pos-_[95] and various pollutant emissions is greater than RPA; that is, v.a_pos-_[95] evaluates the intensity of driving behavior better than RPA.

## Conclusion

This study conducted a thorough analysis of the key influencing factors in Real Driving Emissions (RDE) tests, with the main findings encompassing the following aspects:

The Prominent Role of Driving Behavior on Emissions: The study revealed the direct impact of driving behavior on pollutant emissions, particularly that aggressive driving significantly elevates NO_X_ emissions, while low-temperature conditions markedly increase PN emissions. This discovery underscores the importance of optimizing driving behavior to reduce emissions.

The Complex Influence of Altitude on Emissions: As altitude increases, the CO emission factor notably rises, whereas the NO_X_ and PN emission factors exhibit “N”-shaped and “V”-shaped trends, respectively. This indicates that emission control strategies must be tailored to specific altitudes.

The Significance of the Cold Start Phase: The cold start phase significantly impacts pollutant emission factors during urban driving, particularly with substantial increases in CO and PN emissions. Consequently, improving emission performance during the cold start phase is crucial for reducing overall emissions.

The Correlation between Driving Behavior Intensity and Emissions: By comparing emission data under different driving conditions, the study found a strong positive correlation between driving behavior intensity and CO and PN emission factors. This suggests that evaluating and adjusting driving behavior intensity can effectively control emission levels.

Exploration of Dynamic Parameters and Their Relationship with Emissions: v.apos-[95], as an effective parameter for assessing driving behavior intensity, exhibits a higher correlation with pollutant emissions than other dynamic parameters like RPA, providing a more accurate basis for optimizing driving behavior.

Planned Research Directions:

(1) Further investigation of the relationship between driving behavior and emission factors, including the impact of different acceleration and deceleration patterns on various pollutants.
(2) Examination of the combined effects of altitude and temperature on emission factors, particularly in high-altitude and cold regions.
(3) Study of advanced emission control technologies and their effectiveness in reducing emissions under aggressive driving conditions and in low-temperature environments.
(4) Development of driver training programs and incentives to promote more environmentally friendly driving practices, especially in urban and suburban areas.
(5) Investigation of the impact of traffic congestion and road design on driving behavior and subsequent emissions, with a focus on improving road infrastructure to reduce emissions.

**Table:** 

Nomenclature:			
MAW	moving average window method	VOC	volatile organic compound
CFC	chlorofluorocarbon	a	acceleration
V	velocity	NDIR	non-dispersive infrared spectroscopy
PEMS	portable emission measurement system	GPS	global positioning system
CO	carbon dioxide	PN	particle number
NO_X_	nitrogen oxides	CPC	condensation particle counter
CLD	chemiluminescent detector	OBD	on-board diagnostic
GPS	global positioning system	RDE	real driving emission

## Supporting information

S1 Data Analysis V∙_apos_[95] & RPA. (XLSX)

S2 Data Analysis the RDE driving cycle. (XLSX)

S3 Data Analysis the emission factors. (XLSX)

S4 Data Analysis the impact of cold start on pollutant emissions. (XLSX)
